# Prophylactic Radiotherapy Of MInimally Symptomatic Spinal Disease (PROMISSeD): study protocol for a randomized controlled trial

**DOI:** 10.1186/s13063-023-07850-8

**Published:** 2024-01-12

**Authors:** Robert J. Rothrock, Ahmad Ozair, Maria C. Avendano, Susana Herrera, Haley Appel, Suyen Ramos, Amy K. Starosciak, Daniel S. Leon-Ariza, Muni Rubens, Michael W. McDermott, Manmeet S. Ahluwalia, Minesh P. Mehta, Rupesh R. Kotecha

**Affiliations:** 1 Office 1R203, Department of Radiation Oncology, Miami Cancer Institute, Baptist Health South Florida, Miami, FL 33176 USA; 2 Herbert Wertheim College of Medicine, Florida International University, Miami, FL USA; 3 Miami Neuroscience Institute, Baptist Health South Florida, Miami, FL USA; 4 Bloomberg School of Public Health, Johns Hopkins University, Baltimore, MD USA

**Keywords:** External beam radiotherapy, Prophylactic radiation, Vertebral fracture, Skeletal-related event, Spine lesion, Vertebral metastasis, Bone metastasis

## Abstract

**Background:**

Early palliative/pre-emptive intervention improves clinical outcomes and quality of life for patients with metastatic cancer. A previous signal-seeking randomized controlled trial (RCT) demonstrated that early upfront radiotherapy to asymptomatic or minimally symptomatic high-risk osseous metastases led to reduction in skeletal-related events (SREs), a benefit driven primarily by subgroup of high-risk spine metastasis. The current RCT aims to determine whether early palliative/pre-emptive radiotherapy in patients with high-risk, asymptomatic or minimally symptomatic spine metastases will lead to fewer SREs within 1 year.

**Methods:**

This is a single-center, parallel-arm, in-progress RCT in adults (≥ 18 years) with ECOG performance status 0–2 and asymptomatic or minimally symptomatic (not requiring opioids) high-risk spine metastases from histologically confirmed solid tumor malignancies with > 5 sites of metastatic disease on cross-sectional imaging. High-risk spine metastases are defined by the following: (a) bulkiest disease sites ≥ 2 cm; (b) junctional disease (occiput to C2, C7-T1, T12-L2, L5-S1); (c) posterior element involvement; or (d) vertebral body compression deformity > 50%. Patients are randomized 1:1 to receive either standard-of-care systemic therapy (arm 1) or upfront, early radiotherapy to ≤ 5 high-risk spine lesions plus standard-of-care systemic therapy (arm 2), in the form of 20–30 Gy of radiation in 2–10 fractions. The primary endpoint is SRE, a composite outcome including spinal fracture, spinal cord compression, need for palliative radiotherapy, interventional procedures, or spinal surgery. Secondary endpoints include (1) surrogates of health care cost, including the number and duration of SRE-related hospitalizations; (2) overall survival; (3) pain-free survival; and (4) quality of life. Study instruments will be captured pre-treatment, at baseline, during treatment, and at 1, 3, 6, 12, and 24 months post-treatment. The trial aims to accrue 74 patients over 2 years to achieve > 80% power in detecting difference using two-sample proportion test with alpha < 0.05.

**Discussion:**

The results of this RCT will demonstrate the value, if any, of early radiotherapy for high-risk spine metastases. The trial has received IRB approval, funding, and prospective registration (NCT05534321) and has been open to accrual since August 19, 2022. If positive, the trial will expand the scope and utility of spine radiotherapy.

**Trial registration:**

ClinicalTrials.Gov NCT05534321. Registered September 9, 2022.

**Trial status:**

Version 2.0 of the protocol (2021-KOT-002), revised last on September 2, 2022, was approved by the WCG institutional review board (Study Number 1337188, IRB tracking number 20223735). The trial was first posted on ClinicalTrials.Gov on September 9, 2022 (NCT05534321). Patient enrollment commenced on August 19, 2022, and is expected to be completed in 2 years, likely by August 2024.

**Supplementary Information:**

The online version contains supplementary material available at 10.1186/s13063-023-07850-8.

## Background

Spine metastases are a common occurrence in many solid tumors, with bone being the third most common organ affected by metastatic disease [1]. Spine metastases are a prominent source of cancer-related morbidity and mortality, despite standard of care treatment [2], and the associated pain commonly impairs function and decreases quality of life. High-risk spine metastases, despite initially being asymptomatic or minimally symptomatic, can also result in more debilitating complications, such as pathologic fractures and spinal cord compression [3]. These skeletal-related events (SREs) are frequently addressed with palliative radiation therapy (RT) [4–6]. However, once a patient’s spinal metastases become severely symptomatic or result in SREs, it is significantly more difficult to return the patient to a low baseline pain burden [7]. The current standard of care in patients with minimally symptomatic (non-opioid medication dependent) spine metastases is to continue systemic therapy or observation with medical management for pain and referral for consideration of palliative RT only when the metastases become significantly symptomatic or result in SRE. This regimen in patients with high-risk spine metastasis warrants re-evaluation because adequate symptom reversal is not achieved in the majority of patients treated with the traditional paradigm.

SREs are defined as pathological spinal fractures, spinal cord compression, and the need for interventions, such as RT, interventional procedures, or surgical interventions. SREs significantly impact health-related quality of life in patients with metastatic disease. These events are a measurable primary outcome and have been used as standard primary endpoints in clinical trials investigating therapies for bone metastases [8, 9]. In a recent study, the baseline rate of SREs in patients with metastatic solid tumors with bone metastases was approximately 65% at 1 year, with the median time to SREs being 155 days [10]. SREs also present a socioeconomic burden and lead to significant healthcare utilization and cost [11–13].

Early palliative treatment has been demonstrated to improve the quality of life and even survival for patients with metastatic cancer [6]. Many specialized centers now advocate for earlier integration of RT in a patient’s treatment course, especially with patients living longer with their systemic disease. While several RCTs have evaluated the efficacy and safety of varying RT regimens for managing symptomatic bone lesions, few studies so far have examined the utility of early, upfront RT for the treatment of asymptomatic or minimally symptomatic spine metastases. It is however known that RT can significantly reduce the risk of SREs in patients with solid tumors metastasizing to the bone [14]. A phase 2, multicenter randomized controlled trial recently evaluated the role of early prophylactic RT in patients with high-risk bone metastases, including spine metastases, in patients with polymetastatic disease [14]. In this study, 78 patients with 122 high-risk bone metastases were enrolled and randomized to systemic therapy or observation, in addition to early prophylactic RT. At 1 year, SREs occurred in 1 of 62 lesions (1.6%) in the RT arm and 14 of 49 lesions (29%) in the standard-of-care systemic therapy arm (*P* < 0.001). Specific high-risk features were associated with a significant difference in the time-to-SRE, with most SREs seen in junctional or bulky (> 2 cm) spine disease (*P* = 0.016). Given this intriguing signal, the primary aim of the current randomized trial is to determine whether upfront, early palliative or pre-emptive RT, compared to standard-of-care systemic therapy in patients with high-risk asymptomatic or minimally symptomatic (non-opioid dependent) spine metastases leads to fewer SREs.

## Methods

### Study design and setting

This is a single-center, parallel-arm, prospective, superiority design, randomized controlled trial in patients with asymptomatic or minimally symptomatic high-risk spine metastasis from solid tumors. The study protocol has been reported following the “Standard Protocol Items: Recommendations for Clinical Interventional Trials” (SPIRIT) guidelines along with SPIRIT-Outcomes reporting recommendations (see Supplementary file 1) [15, 16]. This single-center study will be carried out at Miami Cancer and Neuroscience Institutes, Baptist Health South Florida, which represent a hybrid community-academic hospital in Miami, FL, USA (RRID:SCR_023294). Miami Cancer Institute represents one of the largest free-standing cancer hospitals in the state of Florida and has a diverse patient including, particularly of Hispanic or Latino ethnicity [17]. Subjects in this study are randomized to receive either standard-of-care systemic therapy or upfront, early RT to high-risk spine lesions followed by standard-of-care systemic therapy.

### Inclusion criteria

Eligibility criteria for participants in this trial have been informed by prior trials of spine radiotherapy. Included participants must fulfill each of the following inclusion criteria:

1. Histologically confirmed polymetastatic solid tumor, i.e., with > 5 sites of metastatic disease as seen on cross-sectional imaging
2. Asymptomatic or minimally symptomatic (not requiring opioids) spinal metastases that have high risk features as per the Spinal Instability Neoplastic Score (SINS) framework. These include (i) bulky osseus disease sites, i.e., ≥ 2 cm; (ii) junctional spine disease, including the thoracic apex (Occiput to C2, C7-T1, T12-L2, and L5-S1); (iii) posterior element involvement, including interspinous, unilateral, or bilateral facet joints; (iv) compression deformity of > 50% of the vertebral body [3]
3. Performance status of 0–2 as per the Eastern Cooperative Oncology Group (ECOG) scale
4. Age ≥ 18 years
5. Capacity to provide informed consent for trial participation
6. Participants of reproductive potential must agree to utilize an effective contraceptive method. Individuals of childbearing potential must not be lactating or pregnant

### Exclusion criteria

All participants with polymetastatic disease with high-risk spine metastases from solid tumor malignancies are eligible for enrolment in this study, except for the following exclusion criteria:

1. Participant having received RT previously to the intended lesion which precludes the use of RT plan in the current study based on standard tissue tolerance to RT
2. Significant medical comorbidities that preclude the use of RT
3. Participants that are pregnant or lactating
4. Target lesion(s) for spine RT is/are complicated metastases with clinical or radiological features of cord compression or impending pathological fracture
5. Participants having leptomeningeal disease, i.e., tumor metastasis to the arachnoid and pia mater
6. Participants whose entry to the trial will cause unacceptable clinical delays in their planned management

### Study endpoints

The endpoints selected in this trial have been utilized by similar trials [10, 14] and are defined within the time window from the date of randomization to death or 12 months, whichever occurs first. The primary endpoint of the study is the number of SREs per arm, which is defined as pathological fractures, spinal cord compression, or interventions (palliative RT, interventional procedures, or spine surgery) at 12 months. The secondary endpoints are as follows:

1. Surrogates of health care cost, including the number and the duration of hospitalizations for SREs
2. Pain, as assessed using Brief Pain Inventory (BPI) short-form questionnaire
3. Health-related quality of life, as assessed using the Functional Assessment of Cancer Therapy-General (FACT-G) and the 5-level EuroQoL-5D (EQ-5D-5L)
4. Median pain-free survival (PFS), defined as the time from study entry to the start of opioids or death. This definition has been utilized on basis of Rosen et al. [10]
5. Median overall survival (OS), defined as the time from study entry to death or censoring due to follow-up

### Rationale for study instruments and endpoints

This trial is investigating the impact of early, upfront RT on the number of SREs occurring in participants with high-risk spine metastases. The clinical rationale for using SREs as the primary endpoint has been addressed previously and is further expanded here. It has been demonstrated that once a patient’s spinal metastases become severely symptomatic, it is significantly more difficult to return the patient to a low pain burden with treatment compared to when the lesions were less symptomatic [7]. Additionally, in a recent multicenter study that aimed to describe differences in health resource utilization of SREs across Europe and the USA, nearly 25% of reported SREs were found to warrant inpatient hospitalization with a mean length of stay of 18 days [12]. In this work, over 95% of SREs led to procedural management, of which bone RT was the most frequent. Similarly, another investigation demonstrated that nearly a quarter of all SREs warranted an inpatient admission with a mean length of stay of 19.5 days [13]. Furthermore, it has also been demonstrated that efforts to prevent SREs from occurring considerably lead to cost-reduction related to managing SREs, given the substantial economic cost of interventions warranted [11]. Finally, these events also lead to significant utilization of health resources and a major socioeconomic burden [12, 13]. Therefore, on basis of their clinical, economic, and patient-centric utility, SREs have been chosen as the primary endpoint.

Regarding secondary endpoints, the use of median OS as an outcome measure is widely considered standard in oncology. The rationale for other endpoints follows. The use of median PFS has been based as per the key RCT in bony metastases by Rosen et al. [10]. The assessment of pain, and its impact on the participant, is planned for capture through the BPI, a validated self-reported outcome measure [18]. Spinal origin pain has been shown to affect all domains of the individual’s quality of life. BPI short-form, a 17-item scale, captures information regarding pain severity, pain location, chronicity, degree of relief secondary to therapy, use of pain medications, depression, suffering, and perceived availability of relief, among other aspects. BPI’s validity and reliability has been previously demonstrated, and it has been used in several prior RT trials, including the Radiation Therapy Oncology Group (RTOG) 97-14 [18–20]. BPI asks individuals to score their pain for the last week at its “worst,” “least,” “average,” and “now.” The scale is from 0 to 10, with the typical standard deviation for the item “worst pain” being 2.4 in populations with cancer [18]. Thus, a one-point difference in “worst pain” may be considered a minimum clinically important difference (MCID). Mild pain corresponds to a BPI score of 1–4, moderate pain corresponds to a score of 5–6, and severe pain corresponds to a score of 7–10. BPI also asks individuals to estimate other sensory and reactive components on a similar scale of 0–10.

The study also plans to use FACT-G version 4.0 as an instrument to capture the health-related quality of life, given that pain assessment is closely impacted by and impacts the former. This outcome measure has been utilized previously in radiotherapy trials as well, including the RTOG 97-14 [20]. FACT-G was developed and reported by Cella and colleagues in 1993 [21]. It is a 27-item questionnaire, with each item scored on a 5-point Likert scale (from “not at all” to “very much”). The questionnaire captures information across four domains: physical well-being (7 items), social/family well-being (7 items), emotional well-being (6 items), and functional well-being (7 items). The questionnaire has been reported to have high test-retest reliability and validity, and normative data regarding the same are available both from the general US population and in the US cancer population [22]. FACT-G has also been mapped to EQ-5D [23].

EuroQoL 5-Dimensions (EQ-5D) is a widely utilized family of patient-reported instruments designed to capture and value health. These five dimensions are mobility, self-care, usual activities, pain/discomfort, and anxiety/depression. EQ-5D’s use as an outcome measure is recommended by several health technology assessment authorities [24, 25]. The 3-level EQ-5D (EQ-5D-3L) asks individuals to rate each of the five dimensions across three levels, while the EQ-5D-5L, introduced in 2009, does this for five levels [24, 26]. Whereas the FACT-G is a cancer-specific instrument, the EQ-5D is a two-part, generic preference-based outcome measure.

### Assessment of safety

With regard to the assessment of the safety of the intervention, adverse events (AE) will be defined as any untoward medical occurrence associated with the use of an intervention in humans, whether or not considered intervention-related (21 CFR 312.32 (a)). All AEs will have their relationship to study intervention assessed by a clinician, who will examine and evaluate the participant based on temporal relationship and their clinical judgment. Only AEs that are definitely, probably, or potentially related to the protocol treatment will be reported. AEs will be classified according to the NIH Common Terminology Criteria for Adverse Events (CTCAE) version 5.0.

### Power calculation

Power calculation has been performed by a formally trained institutional biostatistician, based on the primary endpoint of the rate of SREs from the date of participant randomization to their death or 12 months follow-up, whichever occurs first, in the two trial arms. SRE, a composite outcome measure, has been defined as a pathological spine fracture, spinal cord compression, or warranting of a procedure (palliative RT, interventional procedures, or spine surgery). SRE is a binary variable, where the event rate may be defined as the number of spine lesions with SRE occurrence divided by the total number of target spine lesions identified. Given the lesion-based analysis in the current trial, occurrence or non-occurrence of an SRE at one vertebra does not impact the status of other spine lesions within the same individual.

Based on institutional records and previous trials, it is estimated that nearly 60–80% of patients may be successfully followed up for at least 1 year. For the standard of care trial arm, where only routine medical management is done, the rate of SRE is known to be nearly 60% within 1 year. Meanwhile, it is known that approximately three fourths of inpatient radiation consultations for painful bone metastases have been reported to lead to palliative RT. Furthermore, prior studies indicate that nearly 60% of RT-targeted spine lesions had been diagnosed ≥ 4 months prior to RT [7]. Therefore, we believe a 60% estimated event rate (as defined above) at 1 year is a conservative estimate. For the investigational trial arm, where early prophylactic RT is being provided to high-risk spine metastases, an SRE rate of 30% is estimated. The efficacy of RT in controlling pain from spine metastases is well-known. Since RT targets and mitigates, to a certain extent, the pathologic effect of a spinal metastases, this leads to reduced development of major bony pain, which is often a causative factor for SREs. For these SRE rates, inclusion of at least 66 individuals with evaluable SRE endpoints in a 1:1 randomization will allow the trial to achieve > 80% power based on a one-sided, two-sample proportion test with significance level of 0.05. Given that the individuals who withdraw prior to the SRE endpoint occurring may not evaluated as per the trial plan; therefore, a per-protocol analysis will be carried out. The limitations of the per-protocol analysis are mitigated by the use of hard endpoints in the current study along with the high morbidity and dismal prognosis of the participants. In order to ensure a minimum of 33 participants are enrolled per trial arm, we plan to over-accrue by 10%, so that the trial is sufficiently powered for the primary endpoint, resulting in a planned sample size of *N* = 74 participants. Additionally, we note that the unit of analysis is the spine lesion, and a small subset of patients may have multiple evaluable lesions (SREs). Since each lesion will be included separately in the analysis, therefore, the final sample size for the primary endpoint may be higher than estimated. We expect to enroll all 74 participants within 2 years.

Furthermore, the investigational arm is unlikely to result in significantly more deaths, since palliation of symptomatic spine metastases is typically performed as part of routine care using RT. This treatment modality is widely utilized and well-established in this patient population and is highly unlikely to have more toxicity.

### Patient recruitment and accrual

Patient enrollment commenced on August 19, 2022, and is expected to be completed in 2 years, likely by August 2024. Potential research participants are identified by the institutional clinical trials staff, the clinical investigator, or a member of the patient’s management team. In cases where the investigator is a member of the treatment team, they review the electronic medical records to determine if a patient may be a suitable participant for the PROMISSED trial. Potential participants contacted by their treating clinician are referred to the principal or sub-investigator or the clinical trials staff. The principal investigator (PI) may also perform chart review for patients with whom they do not have a therapeutic relationship for the limited purpose of identifying eligible patients for the current trial—in such cases, patient contact information is recorded, and these patients are approached later regarding potential enrollment in the PROMISSED trial. During the process where eligible patients are identified for potential participation, the patient may be asked to provide specific health information that is required for the recruitment and enrollment process. The investigator/trials staff may also review parts of their electronic medical records to determine eligibility. For most of the potential participants, the initial contact between research staff and the prospective participant is through either the treatment team, the PI, or the clinical trials staff working in tandem with the treatment team. The recruitment process discussed above presents no more than minimal risk to the privacy of the individuals undergoing screening. Only reasonable and minimal protected health information (PHI) is maintained as part of a screening log.

Once potential participants are identified, definitive eligibility is confirmed as per the inclusion and exclusion criteria specified previously. Informed consent for participation in the trial is obtained by a credentialed study investigator. During the enrollment process, registering staff complete a study-specific eligibility checklist. The staff member who signs the eligibility checklist confirms whether a participant is eligible for study enrollment.

### Randomization, blinding, and allocation concealment

Participants are randomized to either the standard of care (arm 1) or early, upfront RT plus standard of care (arm 2). At the time of randomization, clinicians fill out the Lesion Identification Worksheet (Supplementary file 2: Appendix 2) to document the ≤ 5 high-risk spine metastases per protocol definition to be followed during the protocol in both arms. Once the participant’s eligibility is established, the registration is finalized, and the participants are randomized by the statistician for the study. Randomization is accomplished by the method of a random permuted block and stratified by disease histology (breast and prostate vs. other solid tumors) and planned standard of care treatment (observation vs. systemic therapy). After the treatment arm is determined by randomization, the research coordinator notifies the investigator of the treatment arm within 24 h of randomization. We employ allocation concealment by hiding the allocation sequence from which participants are assigned to the groups until the assignment to prevent selection bias. Both the treating physician and the participant are informed about the treatment they are receiving.

### Study interventions

All participants in the current study receive systemic therapy as per the standard of care, while arm 2 also receives RT. The specifics of RT, target volume, and organ-at-risk delineation and plan evaluation is performed according to institutional standards. For this protocol, total dose and dose fractionation may be delivered at the discretion of the treating radiation oncologist. All techniques, including conventional, 3D conformal RT (3D-CRT), intensity-modulated RT (IMRT), or SRS techniques may be used, with or without image guidance, as deemed appropriate.

Participants undergo RT to each lesion using one of the dose and fractionation regimens presented in Table 1. A variety of dose fractionation schedules including conventional palliative doses as well as SRS schedules are allowed per protocol. After using appropriate immobilization, a simulation is captured for all participants followed by conduct of a CT or MR imaging in the treatment position. The radiation oncologist determines the utilization, if any, of oral or intravenous contrast.

**Table 1:** Dose and fractionation of radiotherapy in the PROMISSED trial

Total dose	Fractions	Dose per fraction	Verification
2000 cGy	5	400 cGy	MV or KV or MR
3000 cGy	10	300 cGy	MV or KV or MR
3000 cGy	5	600 cGy	KV and CBCT or MR
2400 cGy	3	800 cGy	KV and CBCT or MR
2700 cGy	3	900 cGy	KV and CBCT or MR
2400 cGy	2	1200 cGy	KV and CBCT or MR

Supportive strategies for optimal medical care, including medications for acute RT reactions, are given during the trial at the discretion of the treating physician(s) in bounds of the protocol. These supportive therapies are formally documented as concomitant drugs. Any systemic therapies, such as hormone therapy, chemotherapy, immunotherapy, or targeted therapies, may be given based on the treating physician’s discretion. Pain management in order to help the participant come in position for the RT, but not for long-term control, is allowed to reduce voluntary movement. Opioid pain medications may be used, or their use increased, for positioning study participants for RT as required; however, the participants need to return to the baseline level of pain management after completion of RT. Drugs such as alprazolam are permitted if absolutely required to alleviate the participants’ anxiety or for treatment immobilization purposes. Per-protocol treatment is stopped when there is a systemic or local disease progression leading to enrollment in hospice. If any participants lack the ability or resources to get follow-up CT/MR scans or clinical evaluation, this information is recorded.

### Discontinuation of study intervention

Early discontinuation is considered for a patient based on the patient’s and treating physician’s decision for standard of care therapies. Protocol treatment is discontinued when there is systemic or local progression of disease resulting in hospice enrollment.

### Schedule of assessments

The full schedule of assessments is described in Table 2. All included participants have objective confirmation of metastatic disease through either standard of care biopsy of a metastatic lesion or a radiology review documenting metastatic disease. Within 8 weeks (56 days) of trial entry, all participants will have had a (1) FDG-PET/CT scan or CT of the chest, abdomen, and pelvis; (2) MRI of the spine with and without contrast; (3) full medical history including comorbidities, current medications, and performance status; and (4) physical exam including vital signs (O_2_ saturation, blood pressure, heart rate, respiratory rate, and temperature), weight, and height. Following randomization, all participants in both study arms are scheduled for follow-up at 1 month (± 2 weeks), 3 months (± 4 weeks), 6 months (± 8 weeks), 12 months (± 12 weeks), and 24 months (± 16 weeks) and receive the following assessments: clinical evaluation, assessment of performance status, imaging studies, evaluation of AE (CTCAE v5.0) for individuals in arm 2, BPI Short Form, and EQ-5D (Table 3).

**Table 2:** Schedule of assessments in the PROMISSED trial

Assessments*	Pre-enrollment	Baseline	Treatment	1-month F/U^1^	3-month F/U^1^	6-month F/U^1^	12-month F/U^1^	24-month F/U^1^
Informed consent	X							
Eligibility verification	X							
Physical exam	X^2^				X	X	X	X
Medical history	X^2^							
Performance status		X^3^			X	X	X	X
Skeletal related events (SREs)^4^								
Lesion identification worksheet^5^		X						
Numeric pain rating scale (NPRS)	X^6^		X^8^	X^9^	X	X	X	X
Neurological exam	X^6^				X	X	X	X
Imaging of the spine (MRI preferred, acceptable alternates include CT scan or bone scan)	X^7^				X	X	X	X
Documentation of patient’s pain medication^10^	X^6^							
BPI (QL)^11^		X			X	X	X	X
FACT-G (FA)^12^		X			X	X	X	X
EQ-5D (HP)^13^		X			X	X	X	X
Adverse event (AE) evaluation			X	X	X	X	X	X

*All the assessments will come from randomization. Therefore, both arms (arms 1 and 2) have the same timelines

^1^From randomization (times will be the same for intervention and without intervention) treatment. The window for the follow-up visits will be (1) 1 month ± 2 weeks; (2) 3 months ± 1 month; (3) 6 months ± 2 months; (4) 12 months ± 3 months; (5) 24 months ± 4 months

^2^Within 4 weeks before registration. Registration is considered when the eligibility is signed by the investigator

^3^Performance status can be collected before consent

^4^Performed at any point that the patient meets any of the criteria for the protocol-defined SRE

^5^See Supplementary file 2: Appendix 2: Lesion Identification Worksheet

^6^Within 1 week before registration. Please see Supplementary file 2: Appendix 3: Numeric pain rating scale (NPRS) and Supplementary file 2: Appendix 4: Neurological exam. Registration is considered when the eligibility is signed by the investigator

^7^Within 6 weeks before registration. Registration is considered when the eligibility is signed by the investigator

^8^NPRS is collected from the start of treatment up to the 1-month follow-up visit

^9^At home: Daily with cumulative weekly measurements at 1, 2, and 3 weeks; bring to clinic at 1 month from the date of randomization

^10^Concomitant medications will be recorded as follows: All baseline medications will be recorded during treatment, and in the follow-up period, only pain medications will be collected. If the patient is hospitalized, only pain medications will be collected

^11^Please see Supplementary file 2: Appendix 5: Brief Pain Inventory (BPI)

^12^Please see Supplementary file 2: Appendix 6: The Functional Assessment of Cancer Therapy – General (FACT-G)

^13^Please see Supplementary file 2: Appendix 7: EuroQoL EQ-5D (HP)

**Table 3:** Summary of assessments and data collection in the PROMISSED trial

Before treatment start	At 1 month post randomization	At 3, 6, 12, and 24 months post randomization
• FACT-G	• AE Assessment	• AE assessment
• BPI	• NPRS	• NPRS (required at 3 months)
• EQ-5D		• FACT-G
		• BPI
		• EQ-5D

An MRI or bone scan/CT of the spine is obtained for all participants at baseline and at 3, 6, 12, and 24 months after RT to evaluate the tumor response as well as the assess changes in vertebral bone, both subacute and long-term.

All participants in the upfront RT arm (arm 2) are evaluated for AEs according to CTCAE v5.0 and pain score using BPI short form once every five treatment days (typically at the time of their weekly on-treatment visit). Only participants in arm 2 (RT arm) are evaluated for radiation-related AE. In the event of an SRE, the following assessments will be attempted to be completed within 1 week. As it may not be feasible that the research team will be notified of all SREs within 1 week, or a participant may not be able to come to the institution, these assessments shall be considered optional but shall be endeavored for:

- Clinical examination
- Performance status assessment
- Imaging studies
- AE evaluation (CTCAE v 5.0) for arm 2 participants
- BPI Short Form
- EQ-5D-5L

For those participants that cannot come in for follow-up in-person visits or within 1 week of an SRE occurring, a telephonic assessment is considered sufficient, while clinical evaluation and imaging get postponed. Meanwhile, if the participant in this situation gets any of these assessments completed at another institution, efforts are made to obtain those records. If possible, the AE evaluation is also conducted through the telephonic assessment. If the participant in consideration is unable to complete Quality of Life Assessments, research staff are allowed to assist the patient. Furthermore, the BPI Short Form, the NPRS, and EQ-5D-5L in this case may be administered telephonically or directed to the patient through either postal mail, fax, or email by the physician, physician office assistant, or research staff. The mode of sending and receiving questionnaires is determined based on the participant’s preference. If participants cannot be reached through phone calls, the contact information on file will be used for a multipronged approach to get follow-up data.

### Planned statistical analysis

The primary objective of this study is to compare the rate of SREs from the date of randomization to mortality or 1 year, whichever occurs first, between participants receiving standard of care therapies versus participants receiving palliative RT for high-risk spine metastases. It is expected that nearly 60% of the enrolled participants will have at least 1 year of follow-up. The primary endpoint, SRE, has been previously defined as a composite measure including pathological spine fractures, spinal cord compression, or need of spinal interventions (palliative RT, or orthopedic surgery). SRE is a binary variable, with the rate of SRE defined as the number of lesions having SRE divided by the total number of eligible spine lesions. Given that this analysis is lesion-based, not participant-based, therefore, occurrence or non-occurrence at one lesion does not impact the status of other lesions in the same participant. Around 15% of participants may have multiple lesions, for which each lesion gets evaluated as an independent unit of analysis. Meanwhile, unlike the primary objective, the secondary objectives will be evaluated per participant.

A Wilcoxon rank-sum test will be utilized to compare the number of hospitalizations secondary to SREs between the trial arms. For comparing the quality of life and pain level between the trial arms, we will assess at 3 months, 6 months, 12 months, and within 1 week of any SRE (optional). The individual and overall scores derived from the scales described previously will be summarized at these assessment points using means and standard deviations, or medians, and inter-quartile ranges. We will evaluate categorical variables from EQ-5D-5L between the trial arms using Fisher’s test at each time point as well, from which we will determine odds ratios with 95% confidence intervals. We will summarize other non-quantifiable answers (such as treatment received for pain control) in a descriptive fashion. For the score for pain interference on the BPI Short Form scale, we will consider it as the average of the seven interference questions as long as ≥ 4 questions are complete. The mean difference between the trial arms with regard to scores on these scales will be assessed for both statistical and clinical significance. This will be done using Wilcoxon rank-sum tests and established MCIDs. For scales lacking established MCIDs, the “half standard deviation” rule will be applied, wherein the differences of a half standard deviation in the scores between trial arms considered clinically meaningful. After the study is completed, we will plot data at each assessment point, including the number of trial participants in each arm, mean scale scores, and their trends over time. Based on this, we will consider utilizing more exhaustive regression analyses including generalized linear models. We will utilize log-rank tests to compare time-to-event endpoints such as PFS and OS. For toxicity, we will tabulate all AEs at 3, 6, 12, and 24 months post-randomizations in the early upfront RT arm.

### Ethical considerations, monitoring, and data confidentiality

The study protocol (2021-KOT-002) and informed consent documents were submitted to and approved by the WCG institutional review board (WCG IRB, Puyallup, WA, US, Study Number 1337188, IRB tracking number 20223735) on June 25, 2022. The study staff is responsible for ensuring that all institutional requirements necessary to enroll a participant in the study have been completed and protocol amendments are communicated to relevant stakeholders.

The institutional data safety and monitoring committee (DSMC) monitors this clinical trial according to the MCI data and safety monitoring plan. In its oversight capacity independent of study investigators, the DSMC bears responsibility for suspending or recommending this study. DSMC oversight of study conduct includes ongoing review of AE data. The DSMC reviews reports from all audits, site visits, or study reviews pertaining to this clinical trial and takes appropriate action. This study does not have criteria for stopping early. Therefore, no outcomes on data will be reviewed early.

Participant confidentiality and privacy is strictly held in trust by the participating investigators and their staff. The study protocol, documentation, data, and all other information generated are held in strict confidence. No information concerning the study or the data will be released to any unauthorized third party without prior written approval. All research activities are conducted in as private a setting as possible. The study participant’s contact information is securely stored in an electronic database for internal use during the study. Study participant research data, which is for purposes of statistical analysis and scientific reporting, will be transmitted to and stored at MCI. The study data entry and study management systems used by MCI research staff are secured and password protected. At the end of the study, patient data will be archived at MCI.

### Data monitoring and quality assurance

Routine monitoring or audit activities for this study are conducted by authorized personnel under the Office of Research Integrity following current FDA Regulations, ICH GCP guidelines, institutional Standard Operating Procedures (SOPs), IRB procedures, and other government regulations. The general scope of such visits is to inspect study data, including but not limited to regulatory requirements, source documentation, original medical records/files, and CRF completion, as applicable, following a risk-based monitoring approach.

Real-time quality control activities are conducted to evaluate missing study data and trial inconsistencies. Enrollment rates along with extent and accuracy of evaluations and follow-ups are monitored regularly throughout the trial period. Random-sample data quality checks and protocol compliance audits are performed by research staff.

### Study discontinuation and closure

This trial may be prematurely terminated or suspended in case of sufficient reasonable cause, as discussed below. In such circumstances, written notification, including documentation of reasoning behind study suspension or termination, will be provided by the terminating/suspending stakeholder to trial participants, investigators, funding agencies, the IRB, sponsor, regulatory authorities. We will contact study participants, as applicable, and inform them of alterations to the follow-up visit schedule. Reasons that may warrant termination or suspension include, but are not limited to:

- Unexpected, significant, or unacceptable risks to participants are found
- Sufficient efficacy has been demonstrated that it would be unethical to continue the trial
- Substantial protocol deviations have occurred
- Data collected are missing or unevaluable to an extent where the trial’s objectives cannot be assessed
- The primary endpoint is determined to have been met
- Trial intervention is determined to be futile
- A significant change has occurred to trial funding

### Dissemination plan

All efforts will be made to convey the findings of the PROMISSED trial to healthcare professionals, the trial participants, the public, and other relevant stakeholders. In particular, the trial and its key findings are planned to be submitted to national and international conferences for dissemination. Additionally, publication in a peer-reviewed journal will be pursued upon completion of the trial and the drafting of the trial manuscript. Efforts will be made to collaborate with media agencies to disseminate the study’s findings among the public. Various social media platforms will also be utilized to broaden the dissemination regarding the condition studied in the trial and its findings.

## Discussion

Spine metastases represent a debilitating cause of morbidity and mortality for patients with metastatic solid tumors. While RT is frequently used to treat symptomatic bone lesions, the decision on whether radiotherapy should be used for an asymptomatic bone lesion is currently not a standard of care in the absence of randomized controlled trial data. This trial proposes the evaluation of a new treatment paradigm in which spine metastases are treated with upfront RT before they become symptomatic in the outpatient setting or require inpatient admission for pain control or intervention. The current study may help understand the role of early radiation therapy in the treatment of spinal metastases and the results of this RCT will inform practice in patients undergoing palliative care for spine metastases from solid tumors. If positive, the trial will significantly expand the scope and utility of early prophylactic radiotherapy in high-risk spine metastases.

### Supplementary Information

**Additional file 1:** **Supplementary file 1.** SPIRIT 2013 Checklist: Recommended items to address in a clinical trial protocol and related documents

**Additional file 2:** **Supplementary file 2.** Appendices. Appendix 1: Skeletal related events (SRE) form. Appendix 2: Lesion Identification Worksheet. Appendix 3: Numerical pain rating scale (NPRS). Appendix 4: Neurological exam form. Appendix 5: Brief Pain Inventory (BPI). Appendix 6: The Functional Assessment of Cancer Therapy (Fact‐G). Appendix 7: The EuroQoL (EQ‐5D)

## Data Availability

The principal investigator, co-investigator, and statistician for the protocol will have access to and be responsible for all the data for this study. The study dataset will not be published online but may be shared with interested individuals upon reasonable request and all required regulatory approvals.
