# Supplementary material for: Prophylactic Radiotherapy Of MInimally Symptomatic Spinal Disease (PROMISSeD): study protocol for a randomized controlled trial
Source: Trials. 2024 Jan 12;25:41. doi: 10.1186/s13063-023-07850-8 (PMC10785467; doi:10.1186/s13063-023-07850-8)
Supplement: Supplementary file 2 — Additional file 2: Supplementary file 2. Appendices. Appendix 1: Skeletal related events (SRE) form. Appendix 2: Lesion Identification Worksheet. Appendix 3: Numerical pain rating scale (NPRS). Appendix 4: Neurological exam form. Appendix 5: Brief Pain Inventory (BPI). Appendix 6: The Functional Assessment of Cancer Therapy (Fact‐G). Appendix 7: The EuroQoL (EQ‐5D) [file 13063_2023_7850_MOESM2_ESM.pdf]

APPENDIX 1: SKELETAL RELATED EVENTS (SRE) FORM

Miami Cancer Institute
BAPTIST HEALTH SOUTH FLORIDA

Protocol: 2021-KOT-002
Prophylactic Radiotherapy of Minimally Symptomatic Spinal Disease (PROMISSeD Trial)
Skeletal-Related Events (SRE) Assessment Form

Subject Initials: ______________________

Subject ID: ______________________

Visit Date: ______________________

Skeletal-Related Events (SRE) Assessment

How many lesions does this participant have enrolled? ______________

Is the participant currently taking any opioid medication for pain relating to the lesion enrolled? ☐ YES ☐ NO

How many SREs is the participant experiencing? ______________

Please check off the SREs the participant is currently experiencing. *– Select all that apply.*

☐ Pathological fracture
☐ Spinal cord compression
☐ Palliative radiation
☐ Surgery

Please respond to the questions that pertain to the SREs checked off above.

Pathological Fracture

Date of event __ __ / __ __ __ / __ __ __ __ (d d / M M M / y y y y)

Was the participant hospitalized because of this SRE? ☐ YES ☐ NO

If yes, date of hospitalization __ __ / __ __ __ / __ __ __ __ (d d / M M M / y y y y)

Was the SRE identified through imaging? ☐ YES ☐ NO

If yes, date of scan __ __ / __ __ __ / __ __ __ __ (d d / M M M / y y y y)

Is this SRE related to one or more of the lesions enrolled? ☐ YES ☐ NO

Please identify the lesion(s):

1. ________ 2. ________
3. ________ 4. ________
5. ________

Spinal Cord Compression

Date of event	__ __ / __ __ __ / __ __ __ __ (d d / M M M / y y y y)
Was the participant hospitalized because of this SRE?	☐ YES ☐ NO
If yes, date of hospitalization	__ __ / __ __ __ / __ __ __ __ (d d / M M M / y y y y)
Was the SRE identified through imaging?	☐ YES ☐ NO
If yes, date of scan	__ __ / __ __ __ / __ __ __ __ (d d / M M M / y y y y)
Is this SRE related to one or more of the lesions enrolled?	☐ YES ☐ NO
Please identify the lesion(s):	1. ________ 2. ________ 3. ________ 4. ________ 5. ________

Palliative Radiation

Date of event	__ __ / __ __ __ / __ __ __ __ (d d / M M M / y y y y)
Was the participant hospitalized because of this SRE?	☐ YES ☐ NO
If yes, date of hospitalization	__ __ / __ __ __ / __ __ __ __ (d d / M M M / y y y y)
Was the SRE identified through imaging?	☐ YES ☐ NO
If yes, date of scan	__ __ / __ __ __ / __ __ __ __ (d d / M M M / y y y y)
Is this SRE related to one or more of the lesions enrolled?	☐ YES ☐ NO
Please identify the lesion(s):	1. ________ 2. ________ 3. ________ 4. ________ 5. ________

Surgery

Date of event	__ __ / __ __ __ / __ __ __ __ (d d / M M M / y y y y)

Primary reason for surgery *– Select one.*	☐ Severe bone pain<br>☐ Preventative treatment for pathological fracture<br>☐ To treat pathological fracture<br>☐ Other ________________________
Is this SRE related to one or more of the lesions enrolled?	☐ YES ☐ NO
Please identify the lesion(s):	1. _________ 2. _________<br>3. _________ 4. _________<br>5. _________

APPENDIX 2: LESION IDENTIFICATION WORKSHEET

Miami Cancer Institute
BAPTIST HEALTH SOUTH FLORIDA

Protocol: 2021-KOT-002
Prophylactic Radiotherapy of Minimally Symptomatic Spinal Disease (PROMISSeD Trial)
Lesion Identification Worksheet

Subject Initials: ____________________

Subject ID: ____________________

Lesion Identification Worksheet

This worksheet is to document and to follow the ≤5 highest risk bone metastases identified at the time of randomization to receive either standard of care (Arm 1) or radiation (Arm 2).

Highest risk bone metastases definition per protocol: ***(ensure consistency with protocol)***

1. Bulkiest sites of spinal osseous disease ≥ 2 cm
2. Disease at junctional level, including the thoracic apex (Occiput to C2, C7-T1, T12-L2, and L5-S1)
3. Disease with posterior element involvement, including interspinous, unilateral, or bilateral facet joints.
4. Vertebral body compression deformity > 50%

Example:

Lesion location (detailed): left superior acetabulum

Highest risk bone metastases definition (1, 2, 3 or 4): 2

Date of imaging study: 6/11/2017

Image study type (CT, PET/CT, MRI): CT

Best image series/number (s) to identify the lesion: 101

Screenshot of the imaging study that identifies the lesion and its location (optional):

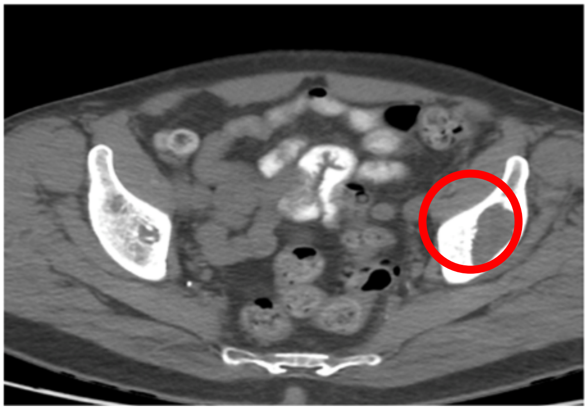

Lesion 1:

Lesion location (detailed): ____________________

____

Highest risk bone metastases definition:
Check one: ☐ 1 ☐ 2 ☐ 3 ☐ 4

Date of imaging study: ____________________
(mm/dd/yyyy)

Image study type: ☐ CT ☐ CT/PET ☐ MRI

Screenshot of the imaging study that identifies the lesion and its location (optional)

____

Best image series/number(s) to identify the lesion:

Lesion 2:

Screenshot of the imaging study that identifies the lesion and its location (optional)

Lesion location (detailed): ______________
____

Highest risk bone metastases definition:
Check one: ☐ 1 ☐ 2 ☐ 3 ☐ 4

Date of imaging study: _________________
(mm/dd/yyyy)

Image study type: ☐ CT ☐ CT/PET ☐ MRI

Best image series/number(s) to identify the lesion:

Lesion 3:

Screenshot of the imaging study that identifies the lesion and its location (optional)

Lesion location (detailed): ______________
____

Highest risk bone metastases definition:
Check one: ☐ 1 ☐ 2 ☐ 3 ☐ 4

Date of imaging study: _________________
(mm/dd/yyyy)

Image study type: ☐ CT ☐ CT/PET ☐ MRI

Best image series/number(s) to identify the lesion:

Lesion 4:

Screenshot of the imaging study that identifies the lesion and its location (optional)

Lesion location (detailed): ______________
____

Highest risk bone metastases definition:
Check one: ☐ 1 ☐ 2 ☐ 3 ☐ 4

Date of imaging study: _________________
(mm/dd/yyyy)

Image study type: ☐ CT ☐ CT/PET ☐ MRI

Best image series/number(s) to identify the lesion:

Lesion 5:

Screenshot of the imaging study that identifies the lesion and its location (optional)

Lesion location (detailed): ______________
____

Highest risk bone metastases definition:
Check one: ☐ 1 ☐ 2 ☐ 3 ☐ 4

Date of imaging study: ________________
(mm/dd/yyyy)

Image study type: ☐ CT ☐ CT/PET ☐ MRI

Best image series/number(s) to identify the lesion:

Additional notes:
____

____

____

Form Completed by: ____ Date: ____________
dd-MMM-yyyy

Site PI Signature: ____ Date: ____________
dd-MMM-yyyy

APPENDIX 3: NUMERICAL PAIN RATING SCALE (NPRS)

Miami Cancer Institute
BAPTIST HEALTH SOUTH FLORIDA

Protocol: 2021-KOT-002
Prophylactic Radiotherapy of Minimally Symptomatic Spinal Disease (PROMISSeD Trial)
Numeric Pain Rating Scale (NPRS) Form

Subject Initials: ______________________

Subject ID: ______________________

Visit Date: ______________________

Numeric Pain Rating Scale (NPRS)

General Information:

- The participant is asked to make three pain ratings, corresponding to current, best, and worst pain experienced over the past 24 hours.
- The average of the 3 ratings was used to represent the patient's level of pain over the previous 24 hours.

Participant Instructions (adopted from McCaffery, Beebe et al. 1989):
"Please indicate the intensity of current, best, and worst pain levels over the past 24 hours on a scale of 0 (no pain) to 10 (worst pain imaginable)."

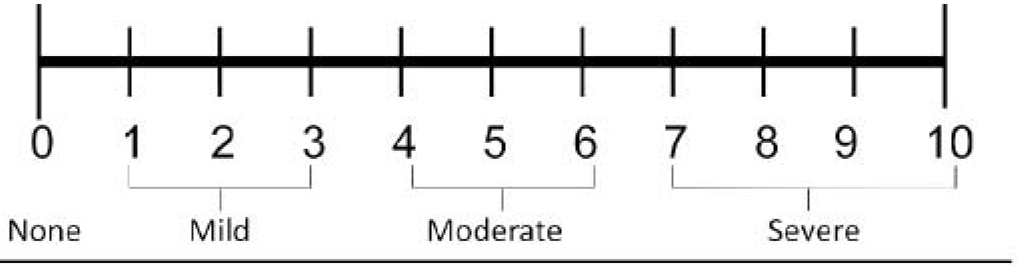

Current Pain Level ____________

Best Pain Level ____________

Worst Pain Level ____________

Average Pain Level ____________
(calculate)

Additional notes: ____

____

Form Completed by: ______________________ Date: ____________
dd-MMM-yyyy

APPENDIX 4: NEUROLOGICAL EXAM FORM

Miami Cancer Institute
BAPTIST HEALTH SOUTH FLORIDA

Protocol: 2021-KOT-002
Prophylactic Radiotherapy of Minimally Symptomatic Spinal Disease (PROMISSeD Trial)
Neurological Exam Form

Subject Initials: ______________________

Subject ID: ______________________

Visit Date: ______________________

Neurological Exam

1. Tenderness over the spine – *Mark the spine level of tenderness on percussion.*

☐ C1 ☐ C2 ☐ C3 ☐ C4 ☐ C5 ☐ C6 ☐ C7 ☐ T1 ☐ T2 ☐ T3 ☐ T4 ☐ T5 ☐ T6 ☐ T7 ☐ T8 ☐ T9 ☐ T10 ☐ T11 ☐ T12

☐ L1 ☐ L2 ☐ L3 ☐ L4 ☐ L5 ☐ Sacrum

2. Radiculopathy – *If present, mark all levels.*

☐ C1 ☐ C2 ☐ C3 ☐ C4 ☐ C5 ☐ C6 ☐ C7 ☐ T1 ☐ T2 ☐ T3 ☐ T4 ☐ T5 ☐ T6 ☐ T7 ☐ T8 ☐ T9 ☐ T10 ☐ T11 ☐ T12

☐ L1 ☐ L2 ☐ L3 ☐ L4 ☐ L5 ☐ Sacrum

3. Muscle strength of the extremities – *Mark 0-5 strength for each.*

Arm Muscles		Strength		Leg Muscles		Strength	
		Right	Left			Right	Left
Deltoid	C5/6			Iliopsoas	L2/3/4		
Biceps	C5/6			Quadriceps	L2/3/4		
Triceps	C6/7/8			Hamstrings	L4/5/S1		
Digit flex (hand grip)	C7/8/T1			Ant Tibialis	L4/L5		
Interossei	C8/T1			Gastrocnemius Soleus	L5/S1/2		

Modified MRC Grade of Muscle Strength

5 Normal strength
5- Equivocal, barely detectable weakness
3 Able to move against gravity without resistance
2 Active movement without gravity

4+ Definite, but slight weakness

4 Able to move against gravity with resistance

1 Flicker or trace of movement

0 No movement observed

4. Sensory change; Pinprick *– Mark normal or decreased.*

	Arm		Trunk		Leg	
	**Right**	**Left**	**Right**	**Left**	**Right**	**Left**
**Normal**						
**Decreased**						

Urinary incontinence (in participants who had initial abnormality or developed new symptoms) *– Select one.*

☐ YES ☐ NO

Anal sphincter tone (in participants who had initial abnormality or developed new symptoms) *– Select one.*

☐ Normal
☐ Decreased
☐ None

Additional notes: ____

____

____

Form Completed by: ______________________ Date: ____________
dd-MMM-yyyy

Site PI Signature: ______________________ Date: ____________
dd-MMM-yyyy

APPENDIX 5: BRIEF PAIN INVENTORY (BPI)

STUDY ID #:__________ DO NOT WRITE ABOVE THIS LINE HOSPITAL #:__________

Brief Pain Inventory (Short Form)

Date: ____/____/____ Time: _______

Name: __________________ __________________ ______________

Last First Middle Initial

1. Throughout our lives, most of us have had pain from time to time (such as minor headaches, sprains, and toothaches). Have you had pain other than these every-day kinds of pain today?

   1. Yes 2. No

2. On the diagram, shade in the areas where you feel pain. Put an X on the area that hurts the most.

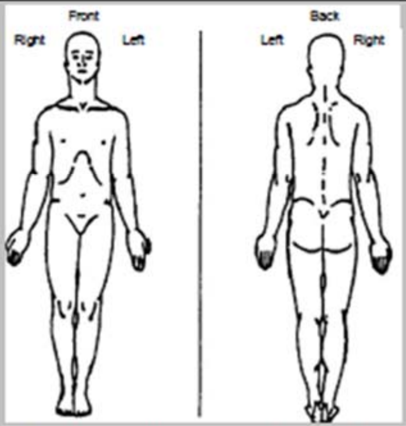

3. Please rate your pain by circling the one number that best describes your pain at its worst in the last 24 hours.

   0 1 2 3 4 5 6 7 8 9 10

   0 = No Pain; 10 = Pain as bad as you can imagine

4. Please rate your pain by circling the one number that best describes your pain at its least in the last 24 hours.

   0 1 2 3 4 5 6 7 8 9 10

   0 = No Pain; 10 = Pain as bad as you can imagine

5. Please rate your pain by circling the one number that best describes your pain on the average.

   0 1 2 3 4 5 6 7 8 9 10

   0 = No Pain; 10 = Pain as bad as you can imagine

6. Please rate your pain by circling the one number that tells how much pain you have right now.

   0 1 2 3 4 5 6 7 8 9 10

   0 = No Pain; 10 = Pain as bad as you can imagine

STUDY ID #: __________ DO NOT WRITE ABOVE THIS LINE HOSPITAL #: __________

Date: ____/____/____ Time: ________

Name: __________________ __________________ ______________

Last First Middle Initial

7. What treatments or medications are you receiving for your pain?

____

____

8. In the last 24 hours, how much relief have pain treatments or medications provided? Please circle the one percentage that most shows how much relief you have received.

0%	10%	20%	30%	40%	50%	60%	70%	80%	90%	100%
No Relief										Complete Relief

9. Circle the one number that describes how, during the past 24 hours, pain has interfered with your:

A. General Activity

0	1	2	3	4	5	6	7	8	9	10
Does not Interfere										Completely Interferes

B. Mood

0	1	2	3	4	5	6	7	8	9	10
Does not Interfere										Completely Interferes

C. Walking Ability

0	1	2	3	4	5	6	7	8	9	10
Does not Interfere										Completely Interferes

D. Normal Work (includes both work outside the home and housework)

0	1	2	3	4	5	6	7	8	9	10
Does not Interfere										Completely Interferes

E. Relations with other people

0	1	2	3	4	5	6	7	8	9	10
Does not Interfere										Completely Interferes

F. Sleep

0	1	2	3	4	5	6	7	8	9	10
Does not Interfere										Completely Interferes

G. Enjoyment of life

0	1	2	3	4	5	6	7	8	9	10
Does not Interfere										Completely Interferes

Copyright 1991 Charles S. Cleeland, PhD
Pain Research Group
All rights reserved

ESTUDIO N°: __________ NO ESCRIBA SOBRE ESTA LINEA HOSPITAL N°: __________

Cuestionario Breve del Dolor (Forma Corta)

Fecha: ____/____/____ **Hora:** _______

Nombre: ________________ ________________ _________________

Primer nombre Apellido Inicial del segundo nombre

1. Todos hemos tenido dolor alguna vez en nuestra vida (por ejemplo, dolor de cabeza, contusiones, dolores de dientes). ¿En la actualidad, ha sentido un dolor distinto a estos dolores comunes?

1. Sí 2. No

2. Indique en el diagrama las zonas dónde siente dolor sombreando la parte afectada. Marque con una cruz la zona que más le duele.

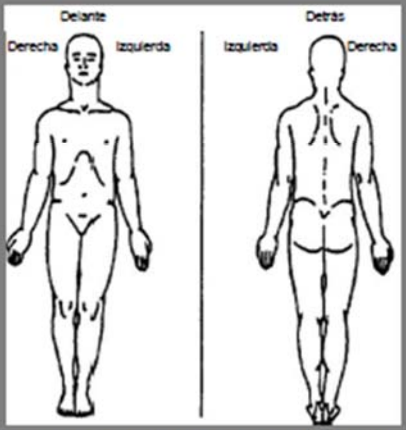

3. Por favor, evalúe su dolor rodeando con un círculo el número que mejor describa la intensidad máxima de su dolor en las últimas 24 horas.

0 1 2 3 4 5 6 7 8 9 10

Ningún Dolor — El Peor Dolor imaginable

4. Por favor, evalúe su dolor rodeando con un círculo el número que mejor describa la intensidad mínima de su dolor en las últimas 24 horas.

0 1 2 3 4 5 6 7 8 9 10

Ningún Dolor — El Peor Dolor imaginable

5. Por favor, evalúe su dolor rodeando con un círculo el número que mejor describa la intensidad media de su dolor.

0 1 2 3 4 5 6 7 8 9 10

Ningún Dolor — El Peor Dolor imaginable

6. Por favor, evalúe su dolor rodeando con un círculo el número que mejor describa la intensidad de su dolor ahora mismo.

0 1 2 3 4 5 6 7 8 9 10

Ningún Dolor — El Peor Dolor imaginable

ESTUDIO N°: __________ NO ESCRIBA SOBRE ESTA LINEA HOSPITAL N°: __________

Fecha: ____/____/____ Hora: _______

Nombre: ________________ ________________ ________________

Primer nombre Apellido Inicial del segundo nombre

7. ¿Qué tratamiento o medicación está recibiendo para el dolor?

8. En las últimas 24 horas, ¿hasta qué punto le han aliviado los tratamientos o la medicación para el dolor? Por favor, rodee con un círculo el porcentaje que corresponda al grado de alivio que ha sentido.

0% 10% 20% 30% 40% 50% 60% 70% 80% 90% 100%

Ningún alivio Alivio total

9. Rodee con un círculo el número que mejor describa hasta qué punto el dolor lo ha afectado en los siguientes aspectos de la vida, durante las últimas 24 horas:

A. Actividad en general

0 1 2 3 4 5 6 7 8 9 10

No me ha afectado — Me ha afectado por completo

B. Estado de ánimo

0 1 2 3 4 5 6 7 8 9 10

No me ha afectado — Me ha afectado por completo

C. Capacidad de caminar

0 1 2 3 4 5 6 7 8 9 10

No me ha afectado — Me ha afectado por completo

D. Trabajo habitual (incluye tanto el trabajo fuera de casa como las tareas domésticas)

0 1 2 3 4 5 6 7 8 9 10

No me ha afectado — Me ha afectado por completo

E. Relaciones con otras personas

0 1 2 3 4 5 6 7 8 9 10

No me ha afectado — Me ha afectado por completo

F. Sueño

0 1 2 3 4 5 6 7 8 9 10

No me ha afectado — Me ha afectado por completo

G. Disfrutar de la vida

0 1 2 3 4 5 6 7 8 9 10

No me ha afectado — Me ha afectado por completo

Copyright 1991 Charles S. Cleeland, PhD
Pain Research Group
All rights reserved

APPENDIX 6: THE FUNCTIONAL ASSESSMENT OF CANCER THERAPY (FACT-G)

FACT-G (Version 4)

Below is a list of statements that other people with your illness have said are important. **Please circle or mark one number per line to indicate your response as it applies to the past 7 days.**

	**PHYSICAL WELL-BEING**	Not at all	A little bit	Some-what	Quite a bit	Very much
GP1	I have a lack of energy	0	1	2	3	4
GP2	I have nausea	0	1	2	3	4
GP3	Because of my physical condition, I have trouble meeting the needs of my family	0	1	2	3	4
GP4	I have pain	0	1	2	3	4
GP5	I am bothered by side effects of treatment	0	1	2	3	4
GP6	I feel ill	0	1	2	3	4
GP7	I am forced to spend time in bed	0	1	2	3	4

	**SOCIAL/FAMILY WELL-BEING**	Not at all	A little bit	Some-what	Quite a bit	Very much
GS1	I feel close to my friends	0	1	2	3	4
GS2	I get emotional support from my family	0	1	2	3	4
GS3	I get support from my friends	0	1	2	3	4
GS4	My family has accepted my illness	0	1	2	3	4
GS5	I am satisfied with family communication about my illness	0	1	2	3	4
GS6	I feel close to my partner (or the person who is my main support)	0	1	2	3	4
Q1	*Regardless of your current level of sexual activity, please answer the following question. If you prefer not to answer it, please mark this box* ☐ *and go to the next section.*					
GS7	I am satisfied with my sex life	0	1	2	3	4

English (Universal)
Copyright 1987, 1997

FACT-G (Version 4)

Please circle or mark one number per line to indicate your response as it applies to the past 7 days.

	EMOTIONAL WELL-BEING	Not at all	A little bit	Some-what	Quite a bit	Very much
GE1	I feel sad	0	1	2	3	4
GE2	I am satisfied with how I am coping with my illness	0	1	2	3	4
GE3	I am losing hope in the fight against my illness	0	1	2	3	4
GE4	I feel nervous	0	1	2	3	4
GE5	I worry about dying	0	1	2	3	4
GE6	I worry that my condition will get worse	0	1	2	3	4

	FUNCTIONAL WELL-BEING	Not at all	A little bit	Some-what	Quite a bit	Very much
GF1	I am able to work (include work at home)	0	1	2	3	4
GF2	My work (include work at home) is fulfilling	0	1	2	3	4
GF3	I am able to enjoy life	0	1	2	3	4
GF4	I have accepted my illness	0	1	2	3	4
GF5	I am sleeping well	0	1	2	3	4
GF6	I am enjoying the things I usually do for fun	0	1	2	3	4
GF7	I am content with the quality of my life right now	0	1	2	3	4

Copyright 1987, 1997

FACT-G (4ª Versión)

A continuación encontrará una lista de afirmaciones que otras personas con su misma enfermedad consideran importantes. **Marque un solo número por línea para indicar la respuesta que corresponde <u>a los últimos 7 días</u>.**

	**<u>ESTADO FÍSICO GENERAL DE SALUD</u>**	Nada	Un poco	Algo	Mucho	Muchísimo
GP1	Me falta energía	0	1	2	3	4
GP2	Tengo náuseas	0	1	2	3	4
GP3	Debido a mi estado físico, tengo dificultad para atender a las necesidades de mi familia.	0	1	2	3	4
GP4	Tengo dolor	0	1	2	3	4
GP5	Me molestan los efectos secundarios del tratamiento	0	1	2	3	4
GP6	Me siento enfermo(a)	0	1	2	3	4
GP7	Tengo que pasar tiempo acostado(a)	0	1	2	3	4

	**<u>AMBIENTE FAMILIAR Y SOCIAL</u>**	Nada	Un poco	Algo	Mucho	Muchísimo
GS1	Me siento cercano(a) a mis amistades	0	1	2	3	4
GS2	Recibo apoyo emocional por parte de mi familia	0	1	2	3	4
GS3	Recibo apoyo por parte de mis amistades	0	1	2	3	4
GS4	Mi familia ha aceptado mi enfermedad	0	1	2	3	4
GS5	Estoy satisfecho(a) con la manera en que se comunica mi familia acerca de mi enfermedad	0	1	2	3	4
GS6	Me siento cercano(a) a mi pareja (o a la persona que es mi principal fuente de apoyo)	0	1	2	3	4
Q1	*Sin importar su nivel actual de actividad sexual, conteste a la siguiente pregunta. Si prefiere no contestarla, marque esta casilla ☐ y continúe con la siguiente sección.*					
GS7	Estoy satisfecho(a) con mi vida sexual	0	1	2	3	4

Spanish (Universal)
Copyright 1987, 1997

FACT-G (4ª Versión)

Marque un solo número por línea para indicar la respuesta que corresponde a los últimos 7 días.

	ESTADO EMOCIONAL	Nada	Un poco	Algo	Mucho	Muchísimo
GE1	Me siento triste	0	1	2	3	4
GE2	Estoy satisfecho(a) de cómo me estoy enfrentando a mi enfermedad	0	1	2	3	4
GE3	Estoy perdiendo las esperanzas en la lucha contra mi enfermedad	0	1	2	3	4
GE4	Me siento nervioso(a)	0	1	2	3	4
GE5	Me preocupa morir	0	1	2	3	4
GE6	Me preocupa que mi enfermedad empeore	0	1	2	3	4

	CAPACIDAD DE FUNCIONAMIENTO PERSONAL	Nada	Un poco	Algo	Mucho	Muchísimo
GF1	Puedo trabajar (incluya el trabajo en el hogar)	0	1	2	3	4
GF2	Mi trabajo me satisface (incluya el trabajo en el hogar)	0	1	2	3	4
GF3	Puedo disfrutar de la vida	0	1	2	3	4
GF4	He aceptado mi enfermedad	0	1	2	3	4
GF5	Duermo bien	0	1	2	3	4
GF6	Disfruto con mis pasatiempos de siempre	0	1	2	3	4
GF7	Estoy satisfecho(a) con mi calidad de vida actual	0	1	2	3	4

Copyright 1987, 1997

APPENDIX 7: THE EUROQOL (EQ-5D)

EQ-5D-5L

Health Questionnaire

English version for the USA

Under each heading, please check the ONE box that best describes your health TODAY.

MOBILITY

I have no problems walking ❑
I have slight problems walking ❑
I have moderate problems walking ❑
I have severe problems walking ❑
I am unable to walk ❑

SELF-CARE

I have no problems washing or dressing myself ❑
I have slight problems washing or dressing myself ❑
I have moderate problems washing or dressing myself ❑
I have severe problems washing or dressing myself ❑
I am unable to wash or dress myself ❑

USUAL ACTIVITIES *(e.g. work, study, housework, family or leisure activities)*

I have no problems doing my usual activities ❑
I have slight problems doing my usual activities ❑
I have moderate problems doing my usual activities ❑
I have severe problems doing my usual activities ❑
I am unable to do my usual activities ❑

PAIN / DISCOMFORT

I have no pain or discomfort ❑
I have slight pain or discomfort ❑
I have moderate pain or discomfort ❑
I have severe pain or discomfort ❑
I have extreme pain or discomfort ❑

ANXIETY / DEPRESSION

I am not anxious or depressed ❑
I am slightly anxious or depressed ❑
I am moderately anxious or depressed ❑
I am severely anxious or depressed ❑

I am extremely anxious or depressed ❑

The best health you can imagine

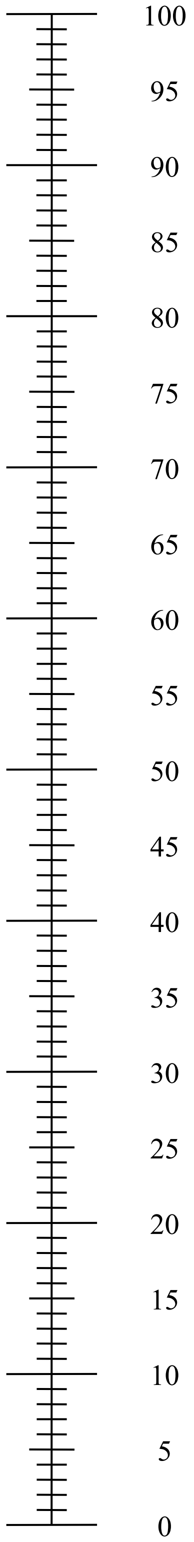

The worst health you can imagine

- We would like to know how good or bad your health is TODAY.
- This scale is numbered from 0 to 100.
- 100 means the best health you can imagine.
  0 means the worst health you can imagine.
- Mark an X on the scale to indicate how your health is TODAY.
- Now, please write the number you marked on the scale in the box below.

YOUR HEALTH TODAY = ☐

EQ-5D-5L

Cuestionario de Salud

Versión en español para los EE. UU.

(Spanish version for the USA)

Debajo de cada encabezamiento, marque UNA casilla, la que mejor describe su salud HOY.

MOVILIDAD

No tengo problemas para caminar ❑

Tengo problemas leves para caminar ❑

Tengo problemas moderados para caminar ❑

Tengo problemas graves para caminar ❑

No puedo caminar ❑

CUIDADO PERSONAL

No tengo problemas para lavarme o vestirme solo/a ❑

Tengo problemas leves para lavarme o vestirme solo/a ❑

Tengo problemas moderados para lavarme o vestirme solo/a ❑

Tengo problemas graves para lavarme o vestirme solo/a ❑

No puedo lavarme o vestirme solo/a ❑

ACTIVIDADES DE TODOS LOS DÍAS *(Ej.: trabajar, estudiar, hacer las tareas domésticas, actividades familiares o actividades de ocio)*

No tengo problemas para realizar mis actividades de todos los días ❑

Tengo problemas leves para realizar mis actividades de todos los días ❑

Tengo problemas moderados para realizar mis actividades de todos los días ❑

Tengo problemas graves para realizar mis actividades de todos los días ❑

No puedo realizar mis actividades de todos los días ❑

DOLOR / MALESTAR

No tengo dolor ni malestar ❑

Tengo dolor o malestar leve ❑

Tengo dolor o malestar moderado ❑

Tengo dolor o malestar intenso ❑

Tengo dolor o malestar extremo ❑

ANSIEDAD / DEPRESIÓN

No estoy ansioso/a ni deprimido/a ❑

Estoy levemente ansioso/a o deprimido/a ❑

Estoy moderadamente ansioso/a o deprimido/a ❑

Estoy muy ansioso/a o deprimido/a ❑

Estoy extremadamente ansioso/a o deprimido/a	❑

- Nos gustaría saber lo buena o mala que es su salud HOY.
- La escala está numerada de 0 a 100.
- 100 representa la <u>mejor</u> salud que se pueda imaginar.
  0 representa la <u>peor</u> salud que se pueda imaginar.
- Por favor haga una X en la escala para indicar cuál es su estado de salud HOY.
- Ahora, por favor escriba en la casilla que encontrará a continuación el número que ha marcado en la escala.

SU SALUD HOY = [ ]

La mejor salud que se pueda imaginar

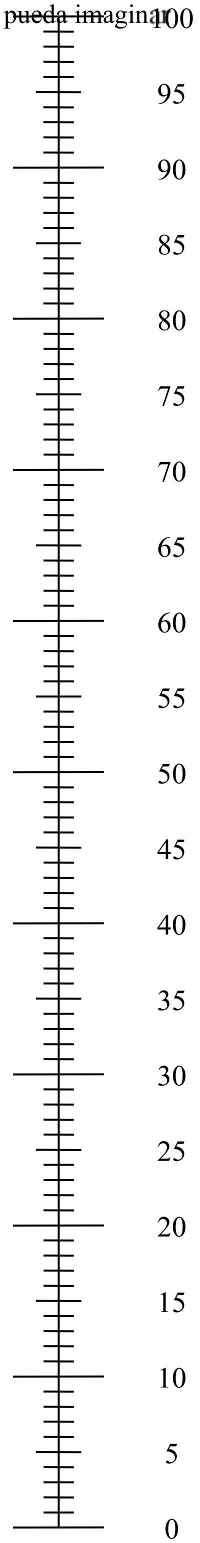

La peor salud que se pueda imaginar
